# Downregulated IL-21 Response and T Follicular Helper Cell Exhaustion Correlate with Compromised CD8 T Cell Immunity during Chronic Toxoplasmosis

**DOI:** 10.3389/fimmu.2017.01436

**Published:** 2017-10-31

**Authors:** Magali M. Moretto, SuJin Hwang, Imtiaz A. Khan

**Affiliations:** ^1^Department Microbiology, Immunology and Tropical Medicine, The George Washington University, Washington, DC, United States

**Keywords:** *Toxoplasma gondii*, T follicular helper cells, exhaustion, IL-21, memory CD8 T cells, latent infection

## Abstract

CD8 T cells are important for maintaining the chronicity of *Toxoplasma gondii* infection. In a *T. gondii* encephalitis susceptible model, we recently demonstrated that CD4 T cells play an essential helper role in the maintenance of the effector response and CD8 T cell dysfunctionality was linked to CD4 T cell exhaustion. However, CD4 T cells are constituted of different subsets with various functions and the population(s) providing help to the CD8 T cells has not yet been determined. In the present study, T follicular helper cells (Tfh), which are known to be essential for B cell maturation and are one of the main sources of IL-21, were significantly increased during chronic toxoplasmosis. However, at week 7 p.i., when CD8 T cells are exhausted, the Tfh population exhibited increased expression of several inhibitory receptors and levels of IL-21 in the serum were decreased. The importance of IL-21 in the maintenance of CD8 T cells function after *T. gondii* infection was further demonstrated in IL-21R KO mouse model. Interestingly, while CD8 T cells from both knockout (KO) and wild-type mice expressed similar levels of PD-1, animals with defective IL-21 signaling exhibited lower polyfunctionality than wild-type controls. This reduced polyfunctional ability observed in CD8 T cells from KO mice was associated with a significant increase in other inhibitory receptors like Tim-3, LAG-3, and 2B4. Furthermore, the animals exhibited greater signs of Toxoplasma reactivation manifested by the reduced number of cysts and increased expression of tachyzoite (replicative form of the parasite) specific genes (*SAG1* and *ENO2*) in the brain. Also, IL-21R KO mice displayed a higher frequency of tachyzoite-infected monocytes in the blood and spleen. Our findings suggest the importance of Tfh and IL-21 during chronic toxoplasmosis and establish a critical role for this cytokine in regulating CD8 T cell dysfunction by preventing the co-expression of multiple inhibitory receptors during chronic parasitic infection.

## Introduction

*Toxoplasma gondii* (*T. gondii*) is an intracellular protozoan parasite of medical significance. The Centers for Disease Control and Prevention has reported that this important foodborne illness affects more than 30 million individuals in the United States with a prevalence rate of over 50% in parts of Europe and Africa (1–3). This opportunist infection is mostly asymptomatic in immunocompetent individuals but flu-like symptoms can sometimes be observed. Reactivation of this latent infection occurs in immunocompromised patients and is very common in AIDS patients with CD4 T cell counts below 200/μL (4). With the introduction of antiretroviral therapy in 1996, the incidence of toxoplasmosis and other opportunistic infections has decreased (5); however, Toxoplasma encephalitis is still the most common central nervous system infections in patients with AIDS in many parts of the world (6, 7) and can have fatal outcomes if not treated properly. In a recent study, latent toxoplasmosis was reported to be associated with the worst neurocognitive disorder in HIV-infected individuals (8). Other serious complications of *T. gondii* infection, such as myocarditis, are also observed in this population (9).

While both CD4 and CD8 T cells have been reported to play a synergistic role in immunoprotection against chronic toxoplasmosis, the effector role is primarily attributed to the CD8 population (10, 11), as depletion of CD8 rather than CD4 T cells results in the mortality of infected animals (10). The importance of effector CD8 T cells in controlling chronic infection has been demonstrated in studies from our laboratory when the functional exhaustion of these cells led to the reactivation of latent disease (12). The synergistic role of CD4 T cells can be defined in terms of the critical help they provide for the maintenance of long-term CD8 T cell immunity against the pathogen (13). Although the requirement for CD4 T cells during chronic toxoplasmosis is well appreciated, the precise role of these cells is not well defined. Studies conducted with viral pathogens, such as HCV, HBV, and LCMV, have reported that CD4 T cell dysfunction observed during the chronic phase of the infection affects CD8 T cell functionality (14–16). Similarly, studies performed in our laboratory have reported that chronic *T. gondii* infection causes CD4 T cell exhaustion, which is attributed to overexpression of transcription factor BLIMP-1. In these studies, we observed that CD4 T cell dysfunction severely compromises CD8 T cell functionality during chronic parasitic infection (17).

CD4 T cells comprise multiple subsets and the individual role of each population in the maintenance of CD8 T cell immunity during *T. gondii* infection has not been defined. T follicular helper cells (Tfh), a specialized subset of CD4 T cells, are essential for germinal centers formation and provide cognate help to B cells (18). These cells are an important source of IL-21, a cytokine that has been described to play a central role in the maintenance of CD8 T cell functionality (19, 20). Furthermore, studies have reported that IL-21R knockout (KO) mice failed to control various intracellular infections (21–23). Previous report from our laboratory has demonstrated that reversal of CD8 T cell exhaustion by blockade of PD-1–PDL-1 interaction led to increased IL-21R expression in chronically infected animals (24). In the current study, we demonstrate a strong induction of Tfh response during *T. gondii* infection, but this subset displayed evidence of exhaustion during the later phase of chronic infection manifested by increased expression of inhibitory receptors like LAG-3 and 2B4. Their dysfunction reduces IL-21 production in the infected host, which compromises CD8 T cell immunity leading to increased reactivation of the infection.

## Results

### *T. gondii* Infection Induces a Strong Tfh Response during Chronic Infection

**Figure 1:**
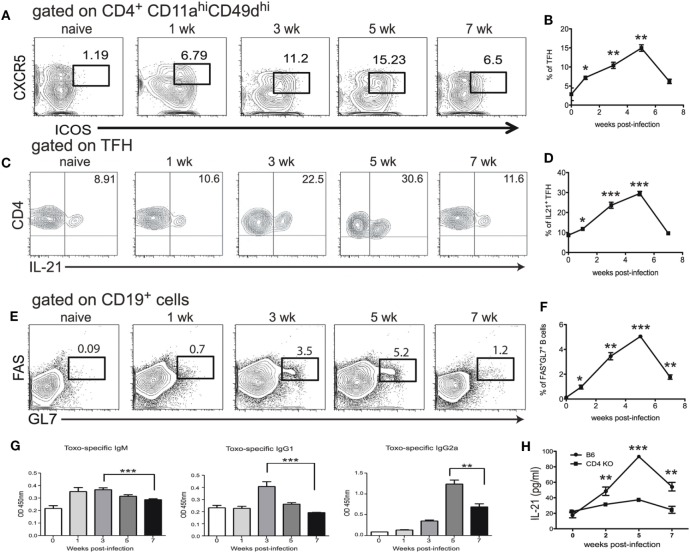
T follicular helper cell (Tfh) response is impaired during chronic toxoplasmosis. C57Bl/6 mice were orally infected with 10 *T. gondii* cysts. Antigen-specific Tfh (ICOS^hi^ CXCR5^hi^) were assessed in the spleen at different time points post infection (weeks 1, 3, 5, and 7 p.i.) **(A,B)**. Plots are gated on antigen-specific CD4 T cells (CD11a^hi^, CD49d^hi^) **(A)**. The graph represents the percentage of Tfh among antigen-specific CD4 T cells **(B)**. IL-21 producing Tfh were measured by intracellular staining in the spleen of infected WT animals at weeks 1, 3, 5, and 7 p.i. and plots are gated on antigen-specific Tfh at different time points post infection (ICOS^hi^CXCR5^hi^CD11a^hi^CD49d^hi^ CD4 T cells) **(C)**. The graph represents the percentage of IL-21-producing cells among Tfh **(D)**. B cells (CD19^+^) were stained for germinal center markers (GL7 and FAS) at the same time points **(E)**. The graph shows the percentage of CD19^+^ cells that express GC markers GL7 and FAS **(F)**. *T. gondii*-specific IgM, IgG1 and IgG2a from sera of infected animals were measured by ELISA at week 1, 3, 5, and 7 p.i. **(G)**. Levels of IL-21 in the serum of infected C57Bl/6 and CD4 KO mice were measured by ELISA **(H)**. Experiments were carried out at least twice and data are representative of one experiment. Student’s *t*-test was used to compare each time points to the naïve controls (*p ≤ 0.05; **p ≤ 0.001; ***p ≤ 0.0001).

Although the expansion of Tfh population during *T. gondii* infection has been reported (25), the role of these cells in the context of CD8 T cell immunity has not been described. In the present studies, at first, a kinetic of the Tfh response during *T. gondii* infection was performed using flow cytometric analysis for ICOS and CXCR5 expression. While ICOS, a potent co-receptor, is highly expressed by these cells, CXCR5 is needed for the Tfh to reach B cell follicles (26, 27). We further validated our phenotypic strategy by confirming that CD4 T cells expressing CXCR5 and ICOS were also PD-1 high (Figure S1 in Supplementary Material). Due to a limited number of defined class II epitopes for *T. gondii* infection (28), we used a surrogate marker strategy that has been published by other groups to identify a broad representation of antigen-specific CD4 T cells (29, 30). We have previously validated this approach by screening CD4 T cells from *T. gondii* infected animals for surrogate marker expression (CD11^a^ CD49^d^) and compared to a known *T. gondii*-specific class II tetramer (17). As shown in Figures 1A,B, a significant increase in Tfh population among the CD11a^hi^CD49d^hi^ CD4 T cell population was observed in the spleen of orally infected mice starting at week 1 p.i. The population peaked at week 5 and declined by week 7 p.i. As Tfh are an important source of IL-21 (31), IL-21 producing Tfh were measured during *T. gondii* infection. As expected, expansion of Tfh population correlated with increased percentage of IL-21 producing Tfh (Figures 1C,D). The role of Tfh in the formation of germinal centers is well established (32) and, consequently, we performed the kinetic of germinal center B cells and *T. gondii*-specific antibody levels in infected animals. As shown in Figures 1E,F, the expansion of a germinal center B cell population expressing high levels of FAS and GL7 is correlated with the increase in Tfh levels. Similarly, *T. gondii*-specific serum antibodies (IgM, IgG1, and IgG2a) also exhibited a comparable pattern (Figures 1E–G). Interestingly, CD4 T cells appear to be the primary source of IL-21 during *T. gondii* infection, as mice lacking CD4 T cells displayed a basal level of this cytokine in the serum (Figure 1H). The decline of the Tfh response at week 7 p.i., a time point at which CD8 T cell exhaustion in *T. gondii*-infected mice becomes apparent (12), suggest an important role for these cells in the maintenance of CD8 T cell immunity against the parasite.

### Upregulation of Inhibitory Receptors by Tfh Population during Chronic Toxoplasmosis

**Figure 2:**
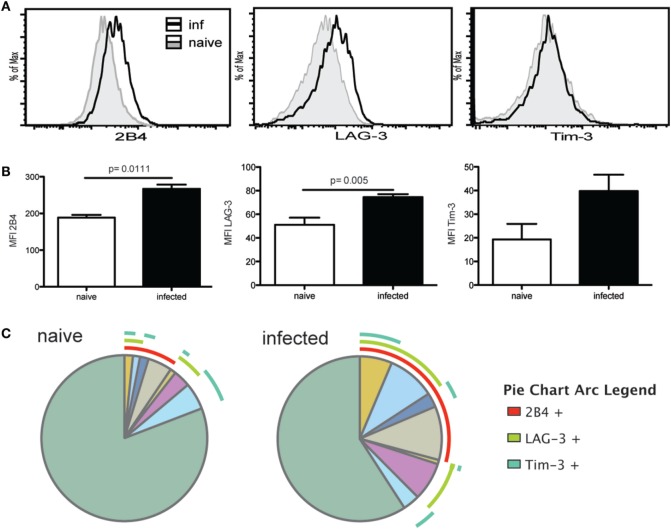
T follicular helper cells (Tfh) exhibit markers of exhaustion during the chronic phase of infection. Expression of exhaustion markers (2B4, Tim-3, and LAG-3) by antigen-specific Tfh at week 7 p.i. and Tfh from naïve animals was analyzed by flow cytometry. Graphs show the overlay of antigen-specific Tfh and Tfh from naïve animals for 2B4, LAG-3, and Tim-3 expression **(A)**. MFI for exhaustion markers is compared between Tfh from naïve controls and antigen-specific Tfh at week 7 p.i. **(B)**. SPICE analysis shows Tfh co-expression pattern for 2B4, LAG-3, and Tim-3 for antigen-specific Tfh at week 7 p.i. and Tfh from naïve mice **(C)**. Experiments were carried out at least twice and data are representative of one experiment.

We have previously reported that chronic *T. gondii* infection results in a decline of CD4 T cell function, which is attributed to an increased expression of inhibitory receptors by the antigen-specific/surrogate marker positive population (17). Surrogate marker-positive Tfh population from week 7 infected mice were evaluated for expression of inhibitory receptors 2B4, LAG-3, and Tim-3. Although PD-1 is an important inhibitory molecule, it is normally expressed by the Tfh population (33) and, thus, evaluation of this marker would have confounded the observations. Interestingly, expression of 2B4 and LAG-3 by surrogate marker-positive Tfh population from *T. gondii*-infected animals was significantly increased compared to the cells from naïve mice (Figures 2A,B). Expression of Tim-3 also appeared to increase compared to naïve Tfh but the difference was not significant. SPICE analysis shows that compared to naïve animals, an increased frequency of Tfh co-expressed multiple inhibitory receptors in KO mice (Figure 2C), which has been previously associated with severe exhaustion of T cells in viral models (34).

### Loss of IL-21 Signaling Increases *T. gondii*-Mediated CD8 Exhaustion

**Figure 3:**
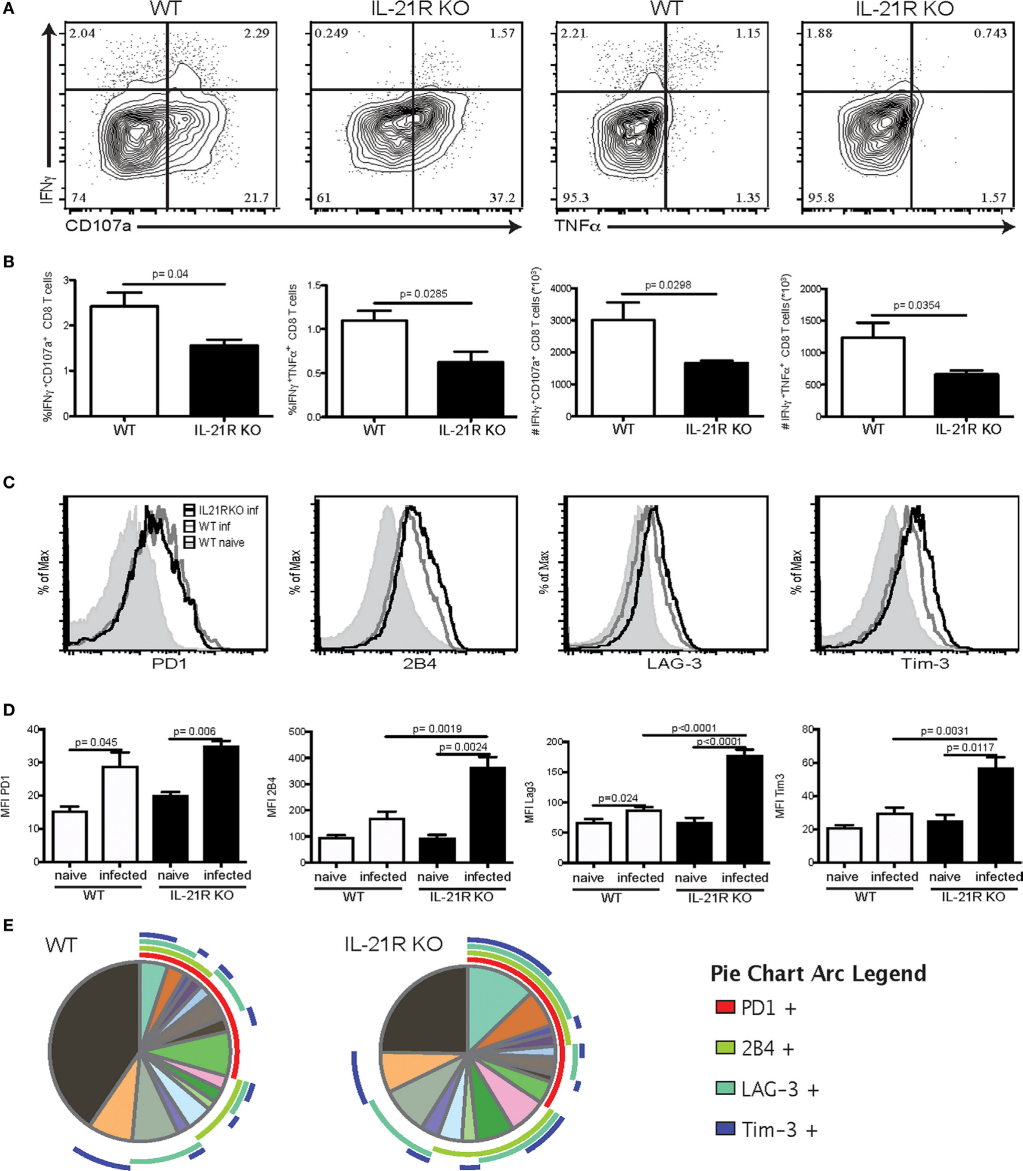

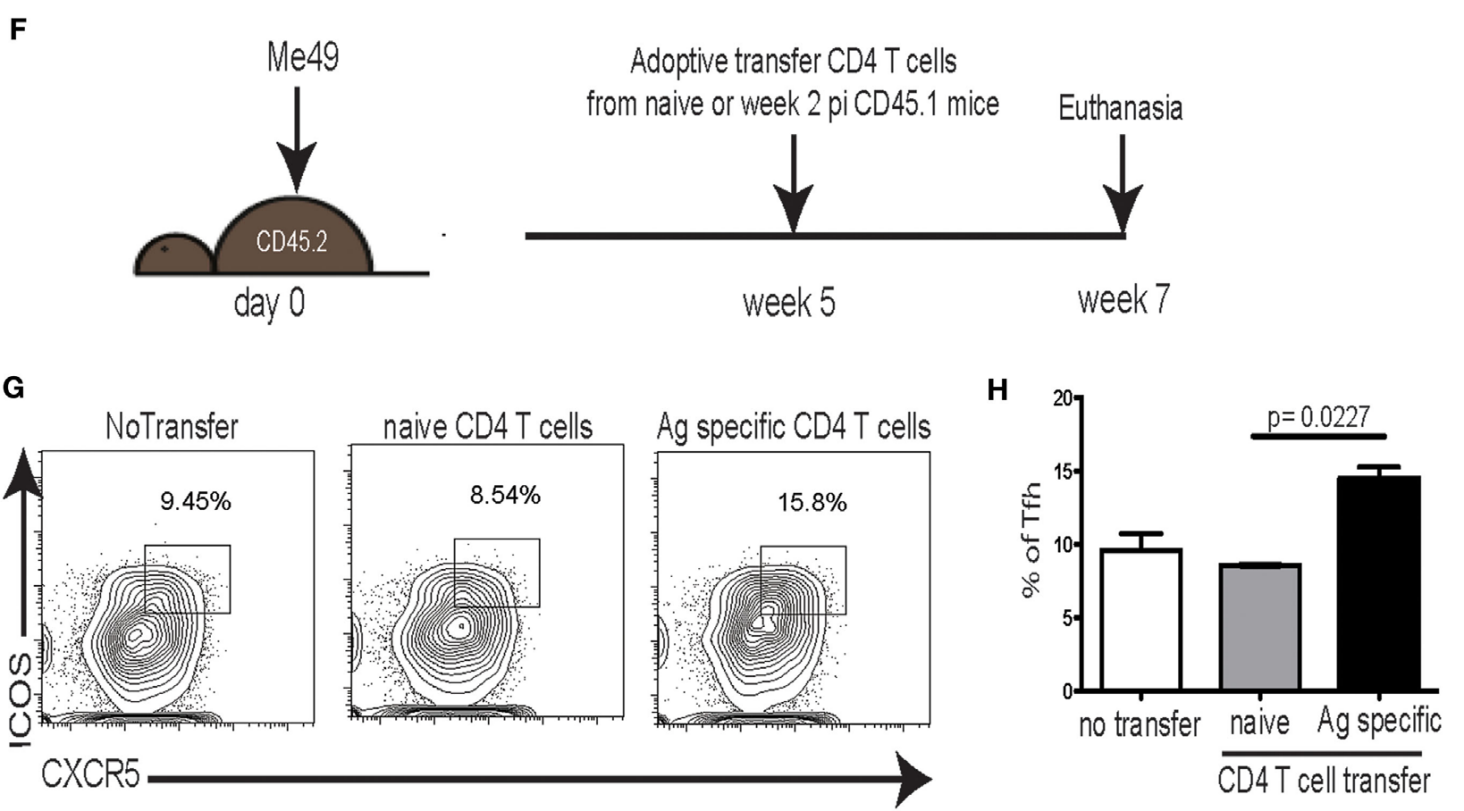
Increased CD8 T cell exhaustion is correlated with loss of IL-21 signaling. WT and IL-21R knockout (KO) animals were infected with 10 *T. gondii* cysts *via* the oral route and CD8 T cell response was analyzed for function and expression of inhibitory receptors at week 7 p.i. **(A–B)** CD8 polyfunctional capability (IFNγ, CD107a, and TNFα) was analyzed after overnight stimulation with Toxoplasma lysate antigen **(A)**. Plots represent CD44^hi^CD11a^hi^ CD8 T cells. Frequency and total number of CD44^hi^CD11a^hi^ CD8 T cells expressing IFNγ/CD107α or IFNγ/TNFα are presented **(B)**. Expression of inhibitory receptors (PD-1, LAG-3, Tim-3, and 2B4) by antigen-specific CD8 T cells (CD44^hi^CD11a^hi^) was measured by flow cytometry analysis **(C)**. Graphs compare the MFI for each marker between WT and IL-21R KO CD8 Tells from naïve and infected animals **(D)**. SPICE pie chart shows the co-expression of the different exhaustion markers for antigen-specific CD8 T cells from WT and IL-21R KO **(E)**. Transfer strategy of *T. gondii*-specific non-exhausted CD4 T cells to mice carrying chronic infection. CD45.2 animals (infected 5 weeks before transfer) received *T. gondii*-specific non-exhausted CD4 T cells (CD4^+^CD11a^hi^CD49d^hi^) isolated at week 2 p.i. or non-specific CD4 T cells (CD4^+^CD11a^lo^CD49d^lo^) from CD45.1 donors (1 × 10^6^ cells/mouse *via* iv. injection) **(F)**. *T. gondii*-specific splenic Tfh were analyzed in the spleen of recipients at week 7 p.i. (2 weeks post-transfer) **(G)**. Bar graph represents the percentage of Tfh among antigen-specific CD4 T cells from the recipient **(H)**. Experiments were carried out at least twice and data are representative of one experiment. Statistical analysis was measured by Student’s *t*-test.

Previous studies from our laboratory have reported that CD8 T cell dysfunctionality during chronic toxoplasmosis (12) is a consequence of CD4 T cell exhaustion (17). In the absence of adequate CD4 T cell help, CD8 T cell population is unable to maintain optimal polyfunctionality, which is needed for controlling the infection. Interestingly, the peak of CD8 T cell dysfunction/exhaustion is observed at week 7 p.i., at the same time point when Tfh population and IL-21 are decreased (Figure 1). Therefore, in the next series of studies, we determined the role of IL-21 in CD8 T cell functionality during chronic Toxoplasmosis using IL-21R KO animals. The polyfunctional activity of surrogate marker-positive (CD11a^hi^CD44^hi^) CD8 T cells, which was also validated by using class I tetramers (17), was measured at week 7 p.i. as described previously (35). As shown in Figures 3A,B, the response of CD8 T cells is downregulated in both WT and IL-21R KO mice, but animals lacking IL-21 signaling exhibited significantly lower polyfunctionality (Figures 3A,B). To determine if increased levels of inhibitory receptors were responsible for the reduced polyfunctionality of CD8 T cells from IL-21R KO mice, expression of PD-1, 2B4, LAG-3, and Tim-3 by surrogate positive CD8 T cells was measured. Interestingly, CD8 T cells from WT and IL-21R KO mice exhibited a similar increase in PD-1 expression (Figures 3C,D). However, as compared to CD8 T cells from WT mice, expression of other inhibitory molecules (2B4, LAG-3, and Tim-3) was significantly increased in mice with impaired IL-21 signaling (Figures 3C,D). Also, a higher frequency of CD8 T cells from IL-21R KO mice co-expressed multiple inhibitory receptors as compared to WT mice (Figure 3E). In a recently published study conducted in our laboratory, we demonstrated that adoptive transfer of surrogate marker positive non-exhausted CD4 T cells prevents CD8 T cell exhaustion in *T. gondii*-infected recipient animals (17). Using the same strategy, in the present report, non-exhausted *T. gondii*-specific CD4 T cells isolated from week 2-infected CD45.1 animals were adoptively transferred to congenic CD45.2 recipients infected 5 weeks earlier. Surrogate marker-specific recipient Tfh (CD45.2) population was evaluated at week 7 p.i. (Figure 3F). As shown in Figure 3H, treatment of recipients with non-exhausted surrogate marker-positive CD4 T cells led to an increase in the percentage of Tfh as compared to the untreated controls or animals that received naïve CD4 T cells. When combined with our published data that non-exhausted CD4 T cell transfer can rescue CD8 T cells response (both in terms of lower inhibitory receptor expression and increased polyfunctionality), the current observations suggest that reversal of CD8 T cell exhaustion is associated with a stronger Tfh response in the recipients.

### Lack of IL-21 Signaling Results in Stronger Reactivation of Latent Toxoplasmosis

**Figure 4:**
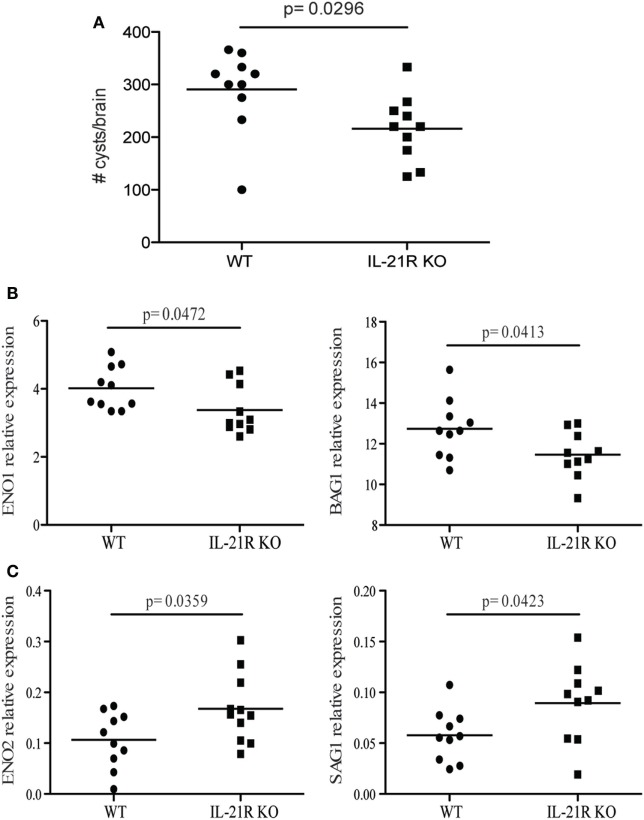
IL-21 is critical for the regulation of *Toxoplasma gondii* reactivation. Cyst numbers were evaluated in the brain of WT and IL-21R knockout (KO) mice at 7 weeks p.i. **(A)**. Relative mRNA expression of tachyzoites (*SAG1* and *ENO2*) **(C)** and bradyzoites (*BAG1* and *ENO1*) **(B)** specific genes in the brain of WT and IL-21R KO mice at 7 weeks p.i. Expression of abovementioned genes was relative to *T. gondii*-specific β actin. Experiments were carried out at three times and data are representative of three combined experiments.

**Figure 5:**
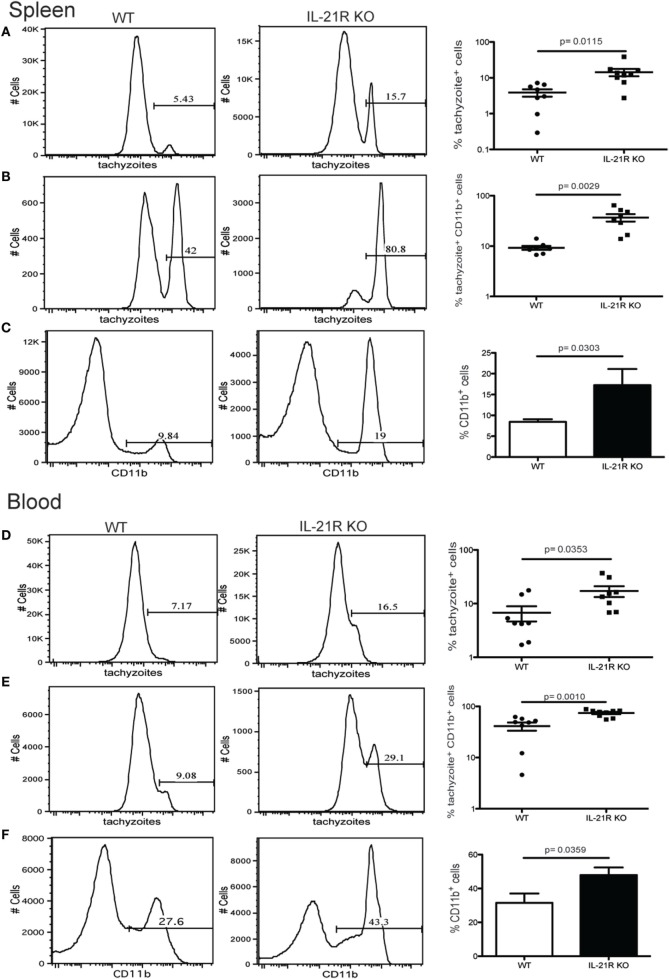
Increase in number of *Toxoplasma gondii*-infected cells after loss of IL-21 signaling. Analysis of *T. gondii*-infected cells in the spleen **(A–C)** and blood **(D–F)** from WT and IL-21R KO-infected animals at week 7 p.i. Plots are gated on total lymphocytes **(A,D)**. Graphs represent the percentage of infected CD11b^+^ **(B,E)**. Percentage of CD11b^+^ cells from infected WT and IL-21R KO mice **(C,F)**. Experiments were carried out at least twice and data are representative of one experiment.

Previous studies from our laboratory have reported that CD8 T cell exhaustion during chronic infection leads to reactivation of latent infection (12). To determine if the lack of IL-21 signaling affects the level of reactivation, brains from KO and WT mice were evaluated for total cyst burden by microscopy and stage-specific antigens by real-time PCR. As shown in Figure 4A, the total number of cysts in the brains of IL-21R KO mice was significantly decreased as compared to WT mice. In support of these observations, KO animals exhibited lower levels of mRNA for bradyzoite-specific genes *ENO1* and *BAG1* (chronic stage) compared to WT mice (Figure 4B). Consequently, the expression of tachyzoite-specific (acute stage) *SAG1* and *ENO2* genes in the brains of IL-21R KO mice were significantly increased. As reactivation of latent toxoplasmosis leads to the dissemination of tachyzoites to other tissues (12), the frequency of tachyzoite-infected cells was also measured in the spleen and blood from infected animals. When compared to WT mice, a significant increase in the number of *T. gondii* infected cells was observed in the spleen and blood of IL-21R KO animals (Figures 5A,D). As a previous study from our laboratory has shown that *T. gondii* preferentially infects leukocytes (12), the number of infected CD11b^+^ cells in the WT versus KO mice was assayed. The significantly increased frequency of infected CD11b^+^ cells was noted in spleen and blood of IL-21R KO animals (Figures 5B,E). Furthermore, IL-21R KO animals exhibited a significantly higher percentage of CD11b^+^ cells in the spleen and blood as compared to WT mice (Figures 5C–F). These findings demonstrate that lack of optimal IL-21 signaling is associated with a greater reactivation of latent toxoplasmosis, which is associated with a more pronounced CD8 T cell dysfunction, suggesting that Tfh and IL-21 are important regulators of CD8 exhaustion during chronic toxoplasmosis.

## Discussion

CD4 T cells play an important synergistic role and provide essential help to CD8 T cells, which is the primary effector population responsible for maintaining the chronicity of infection (10, 13). The CD4 T cells are an essential source of cytokines, which are important mediators of protective immunity against acute Toxoplasmosis (36). Earlier studies from our laboratory showed that in susceptible animals, CD8 T cells get exhausted during the chronic phase of Toxoplasmosis and are unable to prevent the reactivation of latent infection (12). Subsequently, we demonstrated that the underlying causes of CD8 T cell dysfunctionality were linked to CD4 exhaustion (17). In the present study, we demonstrate that Tfh are a critical CD4 T cell subset, which expand in response to *T. gondii* challenge and are an important source of IL-21, the cytokine that regulates CD8 exhaustion during the chronic phase of infection. However, these cells exhibit increased levels of inhibitory receptors during the chronic infection at the same time point when CD8 T cell exhaustion has been reported.

T follicular helper cell population, a specialized subset of CD4 T cells, is critical for providing help to B cells in germinal centers (37, 38) and for their differentiation into plasma cells. Studies conducted with LCMV infection have described the role of IL-21 in the maintenance of CD8 T cell polyfunctionality and regulation of the exhaustion (19, 39). Importantly, the role of CD4 T cells in the maintenance of CD8 T cell immunity against viral infection was linked to their ability to produce IL-21 (39, 40). In recent years, IL-21 has been associated with the development of CD8 T cell immunity against other pathogens (mycobacteria, microsporidia), including HIV infection (22, 23, 41). These findings support our observations that, in the absence of IL-21 signaling, polyfunctional CD8 T cell response against *T. gondii* infection is compromised. Decreased frequency of polyfunctional CD8 T cells in the IL-21R KO animals fails to control chronicity and as a result, an increased number of tachyzoites is observed in the brain, spleen, and blood of these animals. In earlier studies, Stumhofer et al. have reported that IL-21 plays a critical role for optimal antibody production and CD8 T cell numbers during chronic toxoplasmosis (42). Also, similar to our findings, the same group reported an increase in Tfh population during *T. gondii* infection (25). However, in both of these reports, the role of IL-21 and Tfh population was evaluated in the context of antibody production. On the other hand, the data presented in our manuscript emphasize the importance of IL-21 in regulating exhaustion of CD8 T cells and maintaining their polyfunctional ability during chronic toxoplasmosis. Also, it is important to note that reactivation of latent toxoplasmosis in HIV-infected individuals usually occurs when CD4 T cell count falls below 200 cells/ml (4). Therefore, based on our data, the role of Tfh in the reactivation of chronic infection is in an important area of investigation that will need further attention.

As mentioned above, maintenance of polyfunctional CD8 T cells (IFNγ and cytotoxicity) is critical for controlling chronic toxoplasmosis and loss of any of these functions compromises the host immunity against the parasite (43). Studies from our laboratory have shown that during chronic toxoplasmosis, CD8 T cells exhibit a progressive increase in the expression of the inhibitory molecule PD-1, which leads to their exhaustion and loss of function (12). The loss of polyfunctional CD8 T cells causes the reactivation of latent infection manifested by an increase in tachyzoites and decrease of bradyzoites in the tissues. In subsequent studies, it was observed that neutralization of exhaustion markers on CD8 T cells with anti PDL-1 treatment was correlated with an increased expression of IL-21R, suggesting the importance of the cytokine in maintaining functionality (24). Furthermore, increased expression of IL-21R by both CD4 and CD8 T cells was linked to CD40–CD40L intrinsic signaling.

Also, we demonstrated that treatment of toxoplasma-infected animals with surrogate marker-specific non-exhausted CD4 population reversed CD8 exhaustion and controlled the reactivation of latent infection (17). The studies presented in the current manuscript suggest an important role for the Tfh population in keeping chronic Toxoplasmosis under control. This important CD4 T cell subset shows a rise starting 1-week p.i., peaks at 5 and exhibits a decline at week 7 p.i. At week 7 p.i., the Tfh population exhibits increased expression of inhibitory receptors (2B4 and LAG-3) which correlates with decreased IL-21 levels in these animals. It is important to note that this is a time point when CD8 T cell exhaustion becomes apparent (12). Interestingly, adoptive transfer of non-exhausted CD4 T cells to chronically infected mice, which has been reported to rescue CD8 T cell function (17), also increased the survival of the Tfh population (Figure 3G). Interestingly, in our earlier report, the frequency of Tfh cells was increased after anti-PDL-1 treatment (when CD8 T cells were rescued) (24). Thus, the restoration of CD8 functionality following adoptive transfer is likely dependent on increased Tfh survival and higher IL-21 levels. Therefore, in a *T. gondii* model of chronic infection, Tfh appear to be the primary source of IL-21 and exhaustion of these cells compromises CD8 T cell functionality leading to reactivation of the latent infection.

Although exhaustion of Tfh population has not been widely observed, a few reports in tumor models are beginning to emerge (3, 44). In one of these models, increased expression of Tim-3 was implicated in the dysfunctionality of these cells. Our report demonstrates for the first time that Tfh are exhausted during a chronic infection as manifested by increased expression of multiple inhibitory receptors and their reduced ability to produce IL-21. These findings suggest that in mice carrying chronic *T. gondii* infection, Tfh are an important source of IL-21 and their exhaustion likely contributes to CD8 dysfunctionality. The total absence of cytokine signaling in IL-21R KO mice further exacerbates CD8 T cell exhaustion leading to increased reactivation of latent infection in these animals. Although previous studies from our laboratory have implicated PD-1 as the major inhibitory molecule responsible for CD8 T cell exhaustion during chronic toxoplasmosis (12), in the present studies, no significant difference in the expression of PD-1 levels between the CD8 T cells from infected WT and IL-21R KO animals was noted. However, as compared to WT animals a higher frequency of CD8 T cells from KO mice displayed reduced functionality which can be linked to the increased co-expression of multiple inhibitory receptors. These findings are supported by our previous report that, although anti PDL-1 treatment reversed CD8 T cell dysfunction (12), PD-1^hi^ expressing cells could not be rescued. Thus, multiple inhibitory receptor expression may be one of the causes for anti PDL-1 treatment failure to rescue PD-1^hi^ cells. Therefore, it is likely that the role of IL-21 in the regulation of CD8 T cell dysfunction during chronic toxoplasmosis is linked to the expression of multiple inhibitory molecules, which lead to severe exhaustion of these cells and is difficult to reverse. Recent studies related to the blockade of inhibitory receptors on CD8 T cells have demonstrated that cumulative expression of multiple inhibitory receptors limits the ability of these cells to be rejuvenated (45). Thus, the role of IL-21 in regulating multiple inhibitory receptors during chronic Toxoplasmosis is very important and needs to be further investigated.

Overall, based on our observations, we postulate that the increase in the Tfh population during toxoplasmosis occurs at the chronic stage of infection. These cells are an important source of IL-21, which is needed for the maintenance of a robust polyfunctional CD8 T cell response. However, Tfh get exhausted and their decline compromises the levels of IL-21 in the infected animal. The absence of functional IL-21 signaling most likely deprives CD8 T cells of essential help and leads to their increased co-expression of multiple inhibitory receptors. Our findings raise an interesting question about the molecular events that follow the increase in specific inhibitory receptors in the absence of IL-21 signaling. Also, these observations suggest that blockade of PD-1–PDL-1 interaction alone may not be sufficient to prevent reactivation of latent toxoplasmosis. In infectious diseases and cancer models, it has been reported that different inhibitory receptors in addition to PD-1 need to be blocked for optimal reversal of T cell dysfunctionality (46, 47). Based on our findings, the absence of optimal levels of IL-21 during the later phase of chronic *T. gondii* infection may lead to the increased expression of inhibitory molecules other than PD-1 (2B4 and LAG-3) on CD8 T cells. The expression of multiple inhibitory receptors causes severe dysfunctionality in these cells. Whether combinatorial antibody treatment is needed for total and irreversible restoration of CD8 T cell functionality is an important question. Ongoing studies in our laboratory should provide answers to these important questions.

## Materials and Methods

### Animals

7- to 8-week-old C57Bl/6 (CD45.1 and CD45.2), CD4 KO, and IL-21R KO animals were purchased from Jackson Laboratory. All animal studies were carried out in accordance with Institutional Animal Care and Use Committee approved guidelines at The George Washington University.

### Parasites and Infection

All animals were infected *via* oral route with 10 cysts from ME49 *T. gondii* strain prepared from the brain of chronically infected animals. *T. gondii* lysate antigen was prepared from RH strain maintained *in vitro* as previously described (48).

### Cysts Enumeration and Real-time PCR Analysis of Stage-Specific Markers

WT and IL-21R KO animals were infected as described above. Seven weeks p.i., brains were harvested and one-half of the brain was processed for cyst count while other half was flash frozen for subsequent RNA isolation. Cysts were counted by examining 10 µl cell suspension obtained by homogenization in 1 ml PBS using a Dounce homogenizer under a microscope. The total number of cysts in the brain was calculated from an average of six counts from at least three different individuals. RNA was isolated from the flash frozen half of the brain and stage-specific genes were measured by real-time PCR according to standard protocol in our laboratory (12). RNA was isolated using phenol chloroform extraction after homogenization in TRIzol reagent (Thermo Fisher Scientific). Next, cDNA was generated using MMLV reverse transcriptase (Thermo Fisher Scientific) followed by semi quantitative real-time PCR on a CFX96 thermocycler (Bio-Rad). Amplification *of T. gondii*-specific *actin* (5′: TCCCGTCTATCGTCGGAAAG, 3′: CCATTCCGACCATGATACCC), *ENO1 (*5′: GGTATTGATATGCTTATGGTGGAG, 3′: GCGATGTATTTGTATAGTGGTAGG), *ENO2* (5′: CCGTGACAAGGACCAAAC, 3′: ACTCGTTCTTAGTTCCATCG), *SAG1* (5′: ATCGCCTGAGAAGCATCACTG, 3′: CGAAAATGGAAACGTGACTGG) and *BAG1* (5′: GACGTGGAGTTCGACAGCAAA, 3′: ATGGCTCCGTTGTCGACTTCT) genes was conducted with SsoAdvanced Universal SYBR green Supermix (Bio-Rad) at 95°C for 10 min followed by 40 cycles consisting of 15 s at 95°C and 1 min at 6°C. A melt curve was performed to assess the quality of products from amplification. Relative expression of *T. gondii*-specific *actin, ENO1, ENO2, SAG1*, and *BAG1* genes was calculated with ΔCT method.

### Lymphocytes Preparation and Staining

Splenic single-cell suspension was prepared using a standard method from our laboratory (12). Briefly, splenocytes were prepared by mechanical disruption followed by red blood cell lysis. The following antibodies were used for cells surface and intracellular staining: CD4 (GK1.5), CD8β (H35-17.2), CD11a (M17/4), CD49d (R1-2), CD44 (IM7), ICOS (7E.17G9), CXCR5 (L138D7), FAS (SA367H8), GL7 (GL7), IFNγ (XMG1.2), TNFα (MP6-XT22), CD107a (1D4B), PD-1 (RMP-1), LAG-3 (C9B7W), 2B4 (eBio244F4), Tim-3 (B8.2C12), CD45.1 (A20), CD45.2 (104), and CD11b (M1/70). Live/Dead Aqua staining (Thermo Fisher Scientific) and fluorescence-minus-one controls were systematically performed. Cell acquisition was conducted with a Cytek upgraded eight-color FACSCalibur or FACSCelesta cytometer (BD Bioscience) and data were analyzed with FlowJo software. SPICE program provided by M. Roederer (NIH, Bethesda, MD, USA) was used to compute multiple markers analysis.

### Intracellular Staining for Cytokine Detection

For cytokine detection, *in vitro* restimulation was performed for 20 h with 20 µg/ml of *T. gondii* lysate antigen in supplemented Iscove’s DMEM at 37°C with 5% CO_2_. Protein transport inhibitor (Thermo Fisher Scientific) and labeled anti-CD107a were added for the last 4 h of incubation. Following surface staining, cells were fixed and permeabilized using IC Fixation and Permeabilization buffer (Thermo Fisher Scientific) according to manufacturer’s instructions.

IL-21 staining was carried out using an rIL-21R/Fc fusion protein (R&D Systems) followed by PE-conjugated F(ab’)2 goat anti-human Fc (Jackson ImmunoResearch Laboratories) according to a previously published protocol (19).

### Adoptive Transfer of CD4 T Cells

Splenic cell suspension was prepared as mentioned above and CD4 T cells were enriched by negative selection using magnetic EasySep selection kit (Stemcell Technologies) and biotinylated antibodies against B220, CD19, CD11b, NK1.1, Gr1, Ly6G, and Ter19. CD11a^hi^CD49d^hi^ or CD11a^lo^CD49d^lo^ CD4 T cell populations were subsequently sorted using a BD FACS Aria cytometer (purity ≥90%). Mice received 1 × 10^6^ cells *via* intravenous injection.

### Flow Cytometry Detection of *T. gondii* Infected Cells

Single-cell suspension from the spleen and blood were analyzed by flow cytometry after 3-step intracellular staining for tachyzoites. Splenic single-cell suspension was prepared as mentioned above. Peripheral blood was collected in PBS containing 100 U/ml of heparin and partial red blood cell lysis was carried out. Cells were first stained for the surface marker (CD11b) before fixation and permeabilization with Foxp3/transcription factor fixation/permeabilization buffer set (ThermoFisher Scientific). Staining with polyclonal anti-Toxoplasma antibody specific for the tachyzoite stage of the parasite (Abcam) was followed by biotinylated anti-FITC antibody and FITC-conjugated streptavidin staining.

### Statistical Analysis

Statistical significances (*p* < 0.05) for percentages, absolute numbers, MFI, and parasite gene expression from each experiment were evaluated using Student’s *t*-test. Error bars presented in the graphs represent the SD of value between individual mice from each group. All computations were calculated using GraphPad Prism software.

## Ethics Statement

This study was carried out in accordance with the recommendations of the George Washington University Institutional Animal Care and Use Committee under Animal Use Protocol A052.

## Author Contributions

MM, SH, and IK conceived and designed the experiments. MM and SH conducted and analyzed the experiments. MM and IK wrote the manuscript.

## Conflict of Interest Statement

The authors declare that the research was conducted in the absence of any commercial or financial relationships that could be construed as a potential conflict of interest.
