# Supplementary figures and images for: Downregulated IL-21 Response and T Follicular Helper Cell Exhaustion Correlate with Compromised CD8 T Cell Immunity during Chronic Toxoplasmosis
Source: Front Immunol. 2017 Oct 31;8:1436. doi: 10.3389/fimmu.2017.01436 (PMC5671557; doi:10.3389/fimmu.2017.01436)

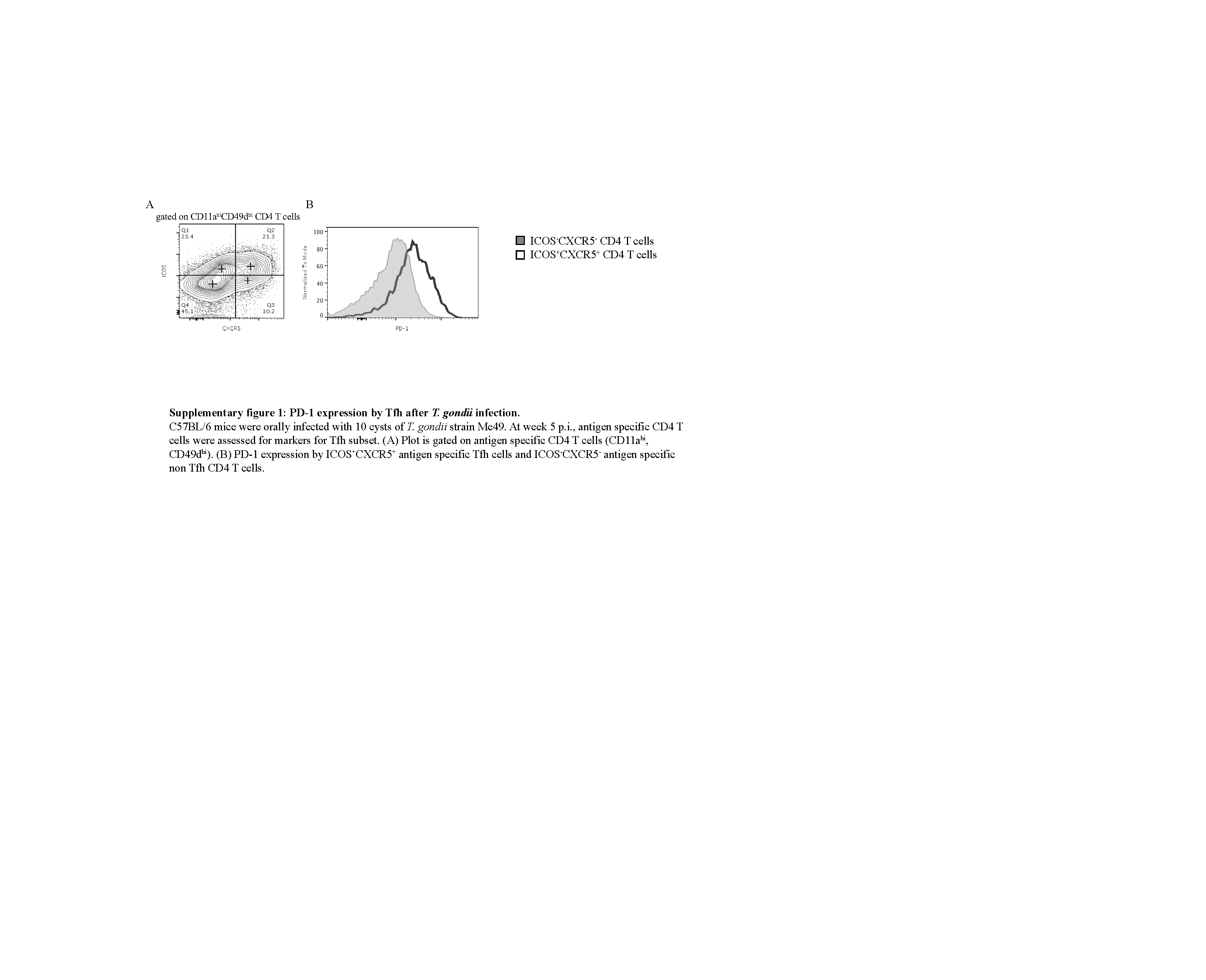

Supplement: Supplementary file 1 [file image_1.tiff]
